# Securing future healthcare environments in a post-COVID-19 world: moving from frameworks to prototypes

**DOI:** 10.1007/s40860-022-00180-7

**Published:** 2022-07-09

**Authors:** Nattaruedee Vithanwattana, Gayathri Karthick, Glenford Mapp, Carlisle George, Ann Samuels

**Affiliations:** 1 Faculty of Science and Technology, Middlesex University, London, UK; 2 Royal Brompton and Harefield Hospital, Uxbridge, UK

**Keywords:** Healthcare systems, Information security frameworks for eHealth and mHealth, Capabilities, Secure remote procedure calls, Service management framework

## Abstract

The deployment of Internet of Things platforms as well as the use of mobile and wireless technologies to support healthcare environments have enormous potential to transform healthcare. This has also led to a desire to make eHealth and mHealth part of national healthcare systems. The COVID-19 pandemic has accelerated the requirement to do this to reduce the number of patients needing to attend hospitals and General Practitioner surgeries. This direction, however, has resulted in a renewed need to look at security of future healthcare platforms including information and data security as well as network and cyber-physical security. There have been security frameworks that were developed to address such issues. However, it is necessary to develop a security framework with a combination of security mechanisms that can be used to provide all the essential security requirements for healthcare systems. In addition, there is now a need to move from frameworks to prototypes which is the focus of this paper. Several security frameworks for eHealth and mHealth are first examined. This leads to a new reference model from which an implementation framework is developed using new mechanisms such as Capabilities, Secure Remote Procedure Calls, and a Service Management Framework. The prototype is then evaluated against practical security requirements.

## Introduction

The COVID-19 pandemic has resulted in major changes in our lives. In an attempt to ensure that hospital facilities do not become overwhelmed, many countries announced lockdown policies to prevent and slow down the transmission of the coronavirus. Furthermore, a number of vaccines have been developed which are being deployed globally to prevent people from getting severely ill; hence, the number of people needing to go to the hospital should be greatly reduced.

Due to the COVID-19 situation, there have also been significant changes in the way healthcare is being delivered. The majority of services have moved to video, telephone, or online sessions. Face-to-face meetings are conducted only when necessary or are unavoidable such as to do medical tests or surgeries. One of the other dramatic changes is the use of medical devices to monitor and record patients’ health parameters. Thus, the use of eHealth and mHealth devices appears to be more accepted going forward, and remote monitoring is now seen as an essential requirement for future healthcare systems.

However, this ongoing transformation has resulted in an intense spotlight being placed on the security of future healthcare environments, because there are now many more ways in which these systems are open to attack, leading to major disruptions in services which will result in longer recovery times and even increased deaths. Also, the security of medical data is an essential requirement mandated by data protection legislation. Article 5(1)(f) of the General Data Protection Regulation (incorporated in the UK Data Protection Act 2018) states that personal data should be “processed in a manner that ensures appropriate security of the personal data, including protection against unauthorised or unlawful processing and against accidental loss, destruction or damage, using appropriate technical or organisational measures” [1].

There are several vulnerabilities that need to be addressed in healthcare environments including: unauthorised access to patient records; ransomware attacks on hospital data; Distributed Denial of Service (DDoS) attacks on hospital Information Technology (IT) infrastructure; the tampering or misuse of hospital equipment; unauthorised access to sensitive data from remote devices; and impersonation of people by others during remote monitoring. This paper addresses these vulnerabilities. It first groups the challenges of securing future healthcare systems into securing five key subsystems. It then looks at frameworks required to meet those goals. It investigates practical mechanisms needed to achieve those goals. Finally, it examines a prototype being developed to test the proposed architecture.

The contributions of this paper are as follows:

1. It attempts to look beyond the present crisis and investigates what the future will hold for healthcare environments including the need to incorporate eHealth and mHealth into national healthcare systems.
2. It presents a comprehensive analysis in terms of Authentication, Authorisation, Accounting and Control (AAAC), information security, network security, and cyber-physical security for this brave new world.
3. It introduces a new flexible capability format which provides security for every object in the system. Using new rules on how these capabilities are used, this paper shows how a system is developed to provide support for AAAC in large-scale environments such as healthcare systems.
4. It then introduces the idea of Transactional Security and a new Secure Transactional Protocol, called the Secure Remote Procedure Call (SRPC), is demonstrated.
5. It introduces a Service Management Framework (SMF) to cope with different healthcare environments as well as user mobility.
6. Building on previous work, it examines an implementation framework.
7. Using this implementation framework, a prototype is developed.
8. The mechanisms introduced here can be applied to other environments and systems.

The rest of the paper is structured as follows: Sect. 2 gives a detailed analysis of security issues in future healthcare systems. Section 3 analyzes related work and the need for developing a new information security framework for eHealth and mHealth systems. Section 4 discusses details of new mechanisms developed in this work. Section 5 implements a proposed framework into a prototype. Section 6 describes the implementation and evaluation of the prototype. Finally, the paper is concluded in Sect. 7.

## Detailed analysis of the security issues in future healthcare systems

As we examine security in healthcare environments, we can divide the security issues into five subsystems.

1. The first requirement is the provision of AAAC for all *human users* including medical staff, patients, retail workers, administrative staff, and visitors. The system should allow users to use the hospital environment simply and intuitively. One way of addressing this is to investigate using mechanisms that support Role-Based Access Control (RBAC) which is a security framework for controlling user access rights to objects in the system based on their roles [2, 3].
2. The second requirement is to protect *devices* from being misused, tampered with, or stolen. This now includes not just medical devices in hospitals and surgeries, but also devices used in the home or by mobile users with eHealth or mHealth functions.
3. The third requirement is the need to protect *digital data* such as the Electronic Health Records (EHRs) of patients. The misuse of EHRs can cause personal as well as economic damage. Hence, it is a legal requirement to protect EHRs as highlighted by the General Data Protection Regulation (GDPR) [1] and the Health Insurance Portability and Accountability Act of 1996 (HIPAA or the Kennedy–Kassebaum Act) [4].
4. There is now also a need to protect *hospital infrastructure*. This is due to the fact that new types of attacks, such as ransomware, are being developed. Ransomware typically attacks (victim) machines in several ways including phishing emails, malicious links, and malvertisings [5, 6]. Network-based attacks such as Denial-of-Service (DoS), Distributed Denial of service (DDoS), and buffer-overflow attacks are on the rise [7, 8].
5. Finally, the presence of COVID-19 increases the need to protect access to certain *physical sites and locations*. This is becoming more important in the UK, where several large hospitals have many departments and access to different parts of the hospital needs to be controlled. Some areas, such as car parks and concourse areas, clearly need to be publicly accessible, while a large number of areas, such as offices and wards, need to have restricted access.

Previous research on security in healthcare usually focused on one or two of the five subsystems discussed above. However, this research looks at a combination of mechanisms that can be used to provide support for all five subsystems.

## Related work and the need for a new information security framework

### Existing studies in security frameworks

Previously, several security frameworks have been developed. They identified security requirements that are needed to be achieved to accomplish essential attributes.

A reusable security requirement template was developed by Firesmith [9, 10]. It defined security as a quality factor that can be divided into underlying subfactors including: Identification, Authentication, Authorisation, Immunity, Integrity, Intrusion Detection, Non-repudiation, Privacy, Security Auditing, Survivability, and Physical Protection. The idea behind reusable security templates is to develop security requirements that potentially can be reused by or extended for any system. Therefore, it can be used to develop an information security framework for healthcare systems.

A security framework based on capabilities [11] (see Sect. 4.1 below) was proposed by Mapp et al. [12]. In this framework, every object in the system is represented using capabilities. This framework can be divided into five layers including: User security layer, Application security, Hypervisor security layer, Transport security layer, and Storage security layer. However, this framework does not specify security requirements for a healthcare system in general as it has been derived from the Firesmith framework [9, 10] and focuses on different Cloud storage functions to develop secure cloud storage.

A joint resource-aware and security framework for the Internet of Medical Things (IoMT)-based remote healthcare systems was proposed by Pirbhulal et al. [13]. This research considered the following requirements: Data Confidentiality, Data Integrity, Data Availability, Data Freshness, Scalability, and Secure Key Distribution, as critical security and privacy requirements for IoMT based healthcare. The framework applied a bio-keys generation mechanism for medical data encryption. The authors argued that this framework is able to assure the security of medical data transmission between patients and physicians as well as decrease the economical healthcare solution.

A hybrid framework for IoT-Healthcare using blockchain technology was proposed by Rathee et al. [14]. They claimed that currently blockchain technology is the best technique to provide secrecy and protection for control systems in real-time conditions. Results of their research showed that this framework offers an 86% success rate, and can prevent wormhole and falsification attacks. However, the drawback of their framework is that hashing of all blocks (nodes) becomes very complicated to predict, since the entire network is maintained by the blockchain.

Research by Yahya in [15] aimed to develop an appropriate security framework for Cloud storage which is one of the main components of a healthcare environment. This framework indicates that security in the Cloud storage can be determined by nine factors: (1) Security policies implementation; (2) Data access protection; (3) Modifications of data stored; (4) Data accessibility; (5) Non-repudiation; (6) Authenticity; (7) Reliability; (8) Accountability; and (9) Auditability.

While the previously discussed frameworks achieve some protection of healthcare data, there is still a need to develop a more comprehensive security framework which provides end-to-end security for healthcare systems. This means security from when healthcare data are collected, then transferred over the network, and stored in both on-site storage and Cloud storage. The need for such comprehensive security has been a driving force behind the development of the new information security framework proposed in this work. It supports the necessary security requirements (Confidentiality, Integrity, Availability, Non-repudiation, Authentication, Authorisation, Accountability, Auditability, and Reliability) [16] and protects every healthcare environment component (devices, hospital infrastructure, digital data, and healthcare data storage) in healthcare systems. Tables 1 and 2 show the comparison of different security frameworks mentioned above in terms of providing security requirements and protecting healthcare environment components. The two tables demonstrate that no existing framework caters for all security requirements and all healthcare environments (see Sect. 2) at the same time.

**Table 1:** Synthesis of security requirements

Requirements	Author				
	[9, 10]	[12]	[13]	[14]	[15]
Confidentiality	*	*	*	*	*
Integrity	*	*	*	*	*
Availability	*	*	*	*	*
Non-repudiation	*			*	*
Authentication	*	*			*
Authorisation	*	*			
Accountability				*	*
Auditability	*			*	*
Reliability		*			*

**Table 2:** Synthesis of healthcare environment components

Components	Author				
	[9, 10]	[12]	[13]	[14]	[15]
Device	*	*			
Hospital infrastructure	*	*			
Digital data	*	*	*	*	
Cloud storage	*	*			*

### Developing a new information security framework and prototype implementation

**Fig. 1:**
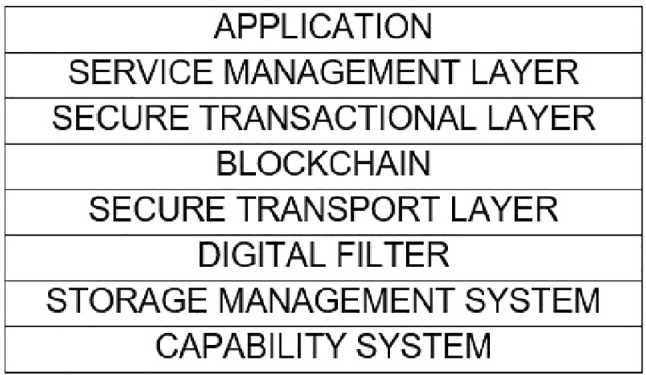
A proposed information security framework for healthcare systems [16]

Previously, an information security framework for healthcare systems was proposed by our authors in [16, 17]. The aim of developing this framework was to provide a complete set of security requirements as follows:

- **Confidentiality:** The assurance that data cannot be viewed by an unauthorised user [18].
- Integrity: Integrity is the assurance that data have not been altered (which includes accidental alteration) in an unauthorised manner [18].
- **Availability:** The assurance that healthcare data will be available and accessible to all authorised users every time it is needed [19]. Non-repudiation: The assurance that an entity cannot deny a previous commitment or action [18].
- **Authentication:** Authentication is the process or action of verifying the identity of a user or process.
- **Authorisation:** Authorisation is a process by which a system determines the security level for access or using resources within the system by each user.
- **Accountability:** Accountability is the process of keeping track of users’ activity while accessing resources in the system.
- **Auditability:** Each organisation in a healthcare system should perform routine security audits to ensure that healthcare data are protected as well as provide policies to comply with the international IT standards.
- **Reliability:** Reliability refers to the ability of a system to provide a consistent intended service most of the time [19]. By considering all the requirements detailed above, a new security framework was proposed. The structure of this new framework is shown in Fig. 1. The various layers are briefly described as follows:
- **Encryption as a Service:** All digital data residing in healthcare environments must be encrypted to protect their confidentiality.
- **Capabilities:** This mechanism allows authorised users to access certain objects in the system.
- **Storage management layer:** This mechanism provides security for the storage of digital data in a healthcare system using encryption techniques, hashing, and replication.
- **Digital filter:** This technique is used to enable more control over who are able to access digital data.
- **Secure transport layer:** Using new protocols such as the Simple Lightweight Transport Protocol (SLTP) [20], this layer provides secure communications while maintaining fast and reliable connections.
- **Blockchain:** A blockchain is a distributed data system where users share a consistent copy of a database and agree on changes by consensus [21]. In healthcare environments, the use of blockchain technology provides the security requirement of non-repudiation and can be used to discover security violations.
- **Secure transactional layer:** This layer provides secure interactions between clients and servers using mechanisms such as the Secure Remote Procedure Call (SRPC).
- **Service management layer:** The Service Management Layer deploys and manages services those are being provided in a healthcare system. Initially, the information security framework for healthcare systems (Fig. 1), was proposed which consists of nine layers, including (1) Encryption as a Service, (2) Capabilities, (3) Storage management, (4) Digital filter, (5) Secure transport layer, (6) Blockchain, (7) Secure transactional layer, (8) Service management layer, and (9) Application. Although, the original framework may strongly secure healthcare systems; however, it will require significant of resources and time which may exceed the PhD timeframe. Moreover, the nature of security in digital environments is never static. New types of security attacks are generated every day, and hence, there will be new studies beyond the current research. Therefore, some new security requirements and mechanisms might be required to completely secure healthcare environments in the future.

### Moving from frameworks to prototypes

Although there has been a lot of work on reference frameworks for healthcare systems, there is a need to move from the framework to a prototype implementation. Since the proposed framework (Fig. 1) consists of many different mechanisms and each mechanism is rather complex to implement. Therefore, developing a viable prototype that clearly embodies every security mechanism, those were mentioned in the previous section, will require a significant effort.

The main purpose of this prototype is to provide an experimental system which contains the necessary functionalities to meet the security requirements that have been identified from our detailed analysis in Sects. 2 and 3.2. New mechanisms are now introduced and developed as key components of the new prototype, namely, Capabilities, Secure Remote Procedure Calls, a Service Management Framework, and an mHealth Secure Storage System. These components are briefly described below:

**Capability system**: Every object in a healthcare system (such as user, device, and healthcare record) will be managed using capabilities to organise access rights which can ensure that only authorised entities will be able to access each object.

**Secure procedure calls:** A Secure Remote Procedure Call (SRPC) has been developed in this work, to protect the remote procedure between the client and the server by encoding the transmitted data. An initial prototype of the SRPC has been implemented and showed a 10% reduction in performance when compared with normal unsafe mechanisms. This is a small price to pay for such a great improvement in security.

**Service management framework**: A Service Management Framework (SMF) is a new approach to managing services in a distributed environment. A simple Service Management Layer (SManL) has already been developed and will be extended as well as integrated into the prototype.

**mHealth secure storage system**: In an mHealth secure storage system, files (e.g., EHRs) are assets of the system. Files can be created, stored, modified, and deleted in the system. The Filesystem in Userspace (FUSE) is used to provide services in controlling how EHRs and other files are accessed, stored, and retrieved. FUSE is connected to the Network Memory Server (NMS) which is a network storage. NMS provides basic functionalities in storing and allocating data blocks.

These mechanisms are now discussed in the next section below.

## New mechanisms in detail

### Capabilities

The term “capability” was originated in 1966 and was coined by Dennis and Van Horn [11]. It refers to a token that permits authorised users to access certain objects in a system. In the Dennis and Van Horn system, there is a pointer to a list of capabilities (C-list). In the C-list, an object in the system is given a name associated with its capability, and the access rights permitted to that object is specified. The type of object name defines the access right in its capability [24].

In this capability-based system, a list of capabilities is initialised when the process is created. When the process makes a request to access an object, the operating system then verifies the capabilities of the process to determine whether it should be granted access or not [25].

In healthcare systems, there is a need to protect the confidentiality and privacy of healthcare records, since there are several people who may have access to them. Only authorised personal with access rights depending on their roles is permitted to access these healthcare records [26].

Capabilities provide several benefits to healthcare systems. Capabilities can be used to provide Role-Based Access Control (RBAC) access for users. Capabilities may not be assigned directly to users; instead, they are assigned to roles, and roles are then assigned to users [27]. Therefore, an access right can be identified by a role, based on job functions of different people in the healthcare system such as doctors, nurses, patients, and researchers.

**Fig. 2:**
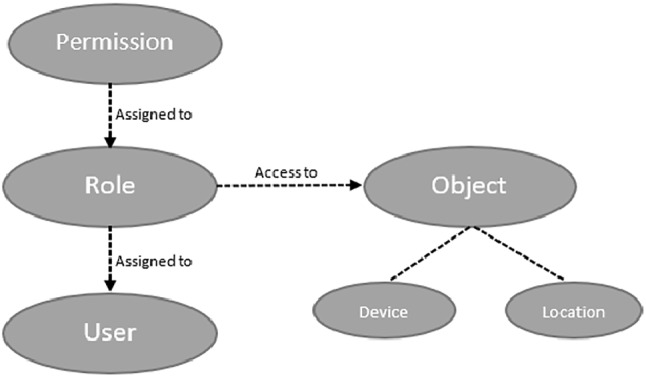
Capability-based system components

A modified role-based capability-based system was developed in this work, based on previous work of Mapp et al. [12]. The main components in the capability-based system (as shown in Fig. 2) include:

- User: Access rights to objects of a user are based on a designated role.
- Role: Roles are identified by job functions.
- Permission: Permissions are clarified by the functionality and responsibility of the job.
- Object: Objects are entities in the system that require protection. Access right to the object may be directly given to the user or may be associated with the user’s role.

#### Capability structure

**Fig. 3:**
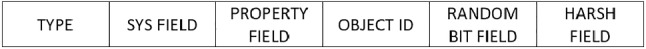
Capability format

The design of the developed capability-based system is based on the flexible approach of IPv6, which uses both unique ID as well as location as a mechanism to allow communications between objects [26]. This approach improves performance and increases security [27]. Every object and its properties can be identified using capabilities. Therefore, it is necessary that capabilities are managed and protected from being created or modified in an unauthorised manner [12, 25]. The format of a capability-based system is shown in Fig. 3.

**Fig. 4:**
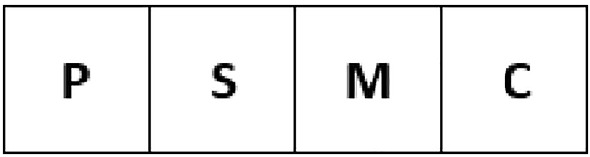
SYS field structure

**1. Type field (8 bits)**: This field is used to specify the type of object capability that is being used. Types include users, digital assets, facilities, etc. The TIMED type is used to indicate a timed-based capability which is only valid for a specified period of time after which the system will refuse to grant any rights to the holder of that capability. In addition, to help administer the system, a special object type known as a Capability List (CL) has been created. The CL is used to group a list of capabilities together. This is explained in more detail below.

**2. SYS field (4 bits):** This field is used to help in managing capabilities. The capability-related fields are given by 4 bits. The structure of the SYS FIELD is shown in Fig. 4:

- **The private or P bit:** This bit is used to restrict the list of people holding the capability. With a private capability, the capability for the object as well as the capability of the subject or the person invoking the object capability must both be presented. Example scenarios include accessing to the hospital main entrance should be a public capability, whereas accessing to a doctor’s office should be a private capability, since only authorised personnel are allowed to access.
- **The system or S bit:** This indicates whether the object involved has been created by the system, or by an application or a user. This means that the capability created by the system cannot be modified or deleted by users or applications. For example, the hospital management creates the hospital concourse capability in the hospital environment. This means that a doctor or a nurse cannot change this capability, and only the hospital management system can change it.
- **The master or M bit:** This bit indicates that the capability was created by a Certificate Authority (CA). This capability is usually created when the object is created. If this bit is not set, it means that this is a proxy capability. Proxy capabilities are derived from master capabilities and cannot be derived from other proxy capabilities. For example, the doctor creates a treatment report file. As the owner of the file, the doctor is given the Master capability for the file which allows him/her to read, write, and delete this file. However, the doctor may want some personnel to be able to read from and write to the file, although, he/she does not want them to delete this file. Hence, the doctor cannot pass them the master capability. Therefore, a proxy capability needs to be created to allow these personnel to read from and write to the file.
- **The change or C bit:** This bit is used to indicate if this capability is changeable. This means that if this bit is set, the proxy capabilities can be derived from the master capability. In a hospital environment, any capabilities for the main entrance or car parks should not be able to change as everyone has to be able to access them.

**3. Property field (12 bits):** This field is used to define the properties of the object. This field relates to properties or functions of the object that the capability represents. For example, the property of doctor can sub-divided into anaesthesia, paediatrics, surgeon, etc.

**4. Object ID (72 bits):** This field is used to uniquely identify the object in the system. Location/ID split network addressing is used [25], where the ID is the standard Extended Unique Identifier (EUI) and uniquely identifies the object.

**Table 3:** Hospital environment capability list

	Object	Doctor	Nurse	Technician	IT Staff
Device	Hand sanitiser Dispenser	C	C	C	C
	Medical cart	R	R	x	x
	Blood pressure meter	R	R	x	x
	X-ray machine	R	x	R	x
	Personal computer	P	P	P	P
Location	Main entrance	C	C	C	C
	Parking	C	C	C	C
	Counselling room	R	R	x	x
	Operating theatre	R	R	x	x
	Laboratory	R	x	R	x
	Informatics unit	x	x	x	R
	Personal office	P	P	P	P
Digital data	EHRs	R	R	x	x
	Clinical photograph	R	R	R	x
	X-ray film	R	R	R	x
	Consent form	R	R	x	x
	Personal correspondences	P	P	P	P
IT infrastructure	Public wifi	C	C	C	C
	On-premise data storage	x	x	x	R

**5. Random bit field (16 bits):** The random bit field provides unforgeability. This field helps to uniquely identify the object. The random bit field is generated after the type field, sys field, property field, and object ID field are created. When proxy certificates are created, a new random field is generated.

**6. Hash field (16 bits):** The hash field is used to allow the detection of the tampering of capabilities. When a capability is created, the type field, sys field, property field, and object ID field are first generated, followed by the random bit field. Finally, these fields are used to generate an SHA-1 hash which is placed in the hash field of the capability.

Two issues which have hindered the widespread use of capabilities are the casual tampering and revocation of capabilities. This capability structure has the hash field that prevents the causal tampering. Moreover, the random bit field also provides the ability to enable easy revocation of capabilities as this can be done by simply changing the random field and recomputing the capability, hence revoking previous versions [25].

**Table 4:** Types supported by SRPC

TYPE	PARAMETER_NO	NUMBER OF BYTES
INT	1	4
U_INT	2	4
SHORT	3	2
U_SHORT	4	2
CHAR	5	1
U_CHAR	6	1
LONG	7	8
U_LONG	8	8
FLOAT	9	4
DOUBLE	10	8
CAPABILITY	11	16
ARRAY	12	Size of array * Size of parameter type
USER_DEFINED_TYPE	13	Variable

**Lists of capabilities**

Lists of capabilities were created as part of the work described in this paper. These lists are used to manage people working at an institution such as a hospital. There are three different types of capability lists:

- **Common:** A common capability list (public capability) belongs to all users in the system.
- **Role-based:** A role-based capability list is assigned to different employees based on their role. For example, doctors are able to access EHRs and medical equipment, or can request a blood test result from a laboratory. Hence, these lists of capabilities can be defined using different role-based types, such as doctors, nurses, hospital staff, etc.
- **Personal:** A personal capability list is used to manage the personal items or spaces of users. This includes access to the personal office, personal correspondences (text messages, emails), etc. Hence, this is a personal capability list will be a list of private capabilities associated with the owner of the object. Table 3 briefly represents how to apply the use of capability list in a real hospital environment. Main persons in hospital environments (e.g., doctor, nurse, and IT staff) hold access rights based on their roles. Devices, locations, digital data, and IT infrastructure are assigned the capability list; Common (C), Role-based (R), and Personal (P) and can only access by authorised capability IDs.

**Capabilities-generating rules**

The capability-based system presented above is very flexible, and so, additional rules are necessary to ensure proper usage when generating capabilities. These rules are given below:

1. A capability is created for an object when an object is being created for the first time. This is called a master capability and the owner of the object will be designated the owner of that capability. Master capabilities must be created by a CA that can issue digital certificates related to this capability.
2. If the change bit in the master capability is set, then it is possible to create proxy capabilities from the master capability.
3. Proxy capabilities must have a new random bit field and a new hash field.
4. Proxy capabilities cannot be created from other proxy capabilities. This rule is necessary to prevent the creation of capabilities by unauthorised persons.
5. System capabilities cannot be generated or changed by users. This rule is needed to protect key entities such as operating systems as well as access to system services.

**Benefits of capabilities**

This section has shown that capabilities can be used in a flexible manner to provide AAAC in the prototype developed and, by extension, for many other environments. For hospital environments, RBAC for humans is sensible and can be easily implemented. However, by requiring all objects to have a capability including devices as well as digital assets such as EHRs, capabilities can be used as a key component of an overarching securing architecture for future healthcare systems.

**Table 5:** Examples of patient records

ID	NAME	Gender	DoB	Contact_No	Address	Emergency_Contact_No
(INT)	(CHAR)	(CHAR)	(UCHAR)	(UCHAR)	(CHAR)	(UCHAR)
1	John Smith	Male	130291	07648832990	23 Langdon Park, London SE166AP	02080342615
2	Rebecca Davis	Female	030978	07466345613	4 Grove Road, Birmingham B145TX	07988743214
3	Nathan Omar	Male	310195	07584421499	Flat 1, 12 Green close, Oxford OX11AA	07828234556

**Table 6:** Encoding of a patient record using SRPC

Field	Type	Value	Add type info	Add type value
Patient record	User defined type = 13	1 - application-specific	No. of entities	7 (made up of 7 fields)
ID	INT = 1	1		
Name	ARRAY = 11		No. of entities	10
	CHAR =5	John Smith (includes space)		
Gender	ARRAY = 11		No. of entities	4
	CHAR = 5	Male		
DoB	ARRAY = 11		No. of entities	6
	UCHAR = 6	130291		
Contact No.	ARRAY = 11		No. of entities	11
	UCHAR = 6	07648832990		
Address	ARRAY = 11		No. of entities	32
	CHAR = 5	23 Langdon Park, London SE16 6AP		
Emergency contact no.	ARRAY = 11		No. of entities	11
	UCHAR = 6	02080342615		

### Secure transactional layer

A secure transactional layer was developed in this work. The purpose behind developing the Secure Transactional Layer is to achieve a secure communication between a client and a server by encoding the data type with its value. Capabilities are also included in this layer to provide authentication and authorisation mechanisms. Furthermore, a Secure Remote Procedure Call (SRPC) was developed, and implemented into the Secure Transactional Layer to secure data transferred between stakeholders and servers.

SRPC is a technique of inter-process communication between client and server to exchange data or execute some instructions. The idea is to use a typed remote procedure call. With this technique, each argument that is passed between client and server must have a defined type as well as a value. The types defined by SRPC is shown in Table 4.

As shown in Table 4, the basic data types (e.g., INT, SHORT) are represented. The CAPABILITY type is used to increase transactional security by allowing the capability structure to be included directly into the SRPC, thus allowing AAAC in every SRPC call. The ARRAY type is used to represent a collection of similar objects. Finally, the USER DEFINED TYPE is used to allow users to define their own secure data passing structures between clients and servers. With this type, the programmer must provide the SRPC routines to encode and decode the USER DEFINED TYPE.

Since SRPC supports an idea of type array, this is an improvement of a traditional RPC where type information is not passed in the RPC call, and hence, only the interface definition of the call is used to interpret the arguments. This, however, can be easily abused as in the case of buffer flow attacks that plague Web-Servers. SRPC, therefore, enables the endpoint receiving RPC calls to check whether the correct types and value have been sent before servicing request.

In addition, this approach can detect changes in the data due to human error, since it is possible to check that both the type and value passed are correct. This is therefore good for configuration systems where human error is quite common.

Table 5 shows the various data types associated with patient records and Table 6 shows a use of SRPC to encode a patient record.

**Fig. 5:**
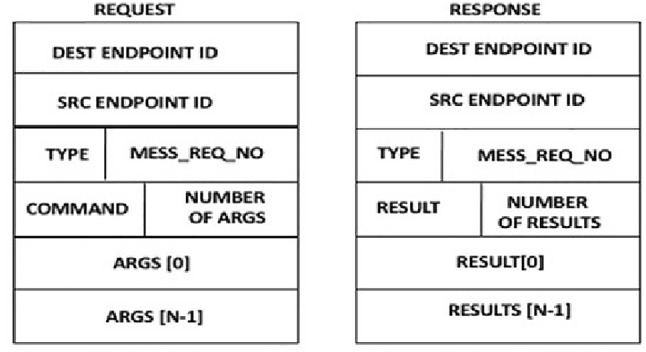
SRPC format

The fields of the SRPC format (Fig. 5) are detailed below:

- **DEST ENDPOINT ID (64 bits):** The communication endpoint of the destination. This uniquely identifies the communication endpoint of the remote end. This is defined using DEST IPV4/IPV6 ADDRESS, DEST TCP/UDP port. Extra bytes must be set to zero.
- **SRC ENDPOINT ID (64 BITS):** The communication endpoint of the source. This uniquely identifies the communication endpoint of the local end. This is defined using SRC IPV4/IPV6 ADDRESS, SRC TCP/UDP port.
- **TYPE (8 BITS):** The type of message: REQUEST = 1, REPLY = 2.
- **MESSAGE_REQ_NO (24 BITS):** The message sequence message number. This must be the same for the request and subsequent response.
- **COMMAND (16 BITS):** The command being asked to be executed by the client. This is an application-specific parameter.
- **RESULT (16 BITS):** The result of executing the command. This again is an application-specific parameter.
- **NUMBER OF ARGS:** The number of arguments in the request.
- **NUMBER OF RESULTS:** The number of result parameters in the response.
- **ARGS[0] — ARGS[N-1]:** The arguments of the request.
- **RESULT[0] — RESULTS[N-1]:** The result parameters of the response. The argument and result parameters are specified using SRPC types, as shown in Table 4.
- **Benefits of SRPC** SRPC allows a much more secure transactional environment to be developed which makes sure that clients and servers can interact securely. In addition, the use of the capabilities means that SRPC allows AAAC to be effective not just for people and devices but also for clients and servers.

### Service management layer

The Service Management Layer (SManL) was developed to manage services by specifying the functions of these services and requirements needed to run them. There is an increasing need to deploy services in different types of networking environments and on many different types of hardware. However, with the deployment of Cloud systems in which servers run in a virtualised environment, multiple services from different domains may share the same hardware system. Furthermore, because of the large amount of data being generated in healthcare environments, Cloud systems are increasingly being used to store and process health data [28]. As discussed in Sect. 1 above, there is a legal requirement to always keep EHRs safe. Hence, the security challenges of using Cloud services for healthcare environments must be addressed to ensure that patients, hospital staff, and visitors are safe.

The challenges can be articulated as follows:

1. A secure execution environment: there is a need to ensure that services are not hosted on unsafe Cloud hardware and Cloud servers are not corrupted by malicious or badly implemented servers.
2. It is necessary to be able to work out the best place to run a service at any point in time. This may be affected by many factors including location, cost, QoS, and security requirements.
3. There must be the ability to securely transfer services between Clouds. A new security protocol, namely, the Resource Allocation Security Protocol (RASP) [29], was proposed to secure service migration over Cloud infrastructure. It supports mobile services to ensure that transfer of resources between different Cloud environments is safe. Hence, the development of RASP is being explored.

**Service management framework (SMF)**

**Fig. 6:**
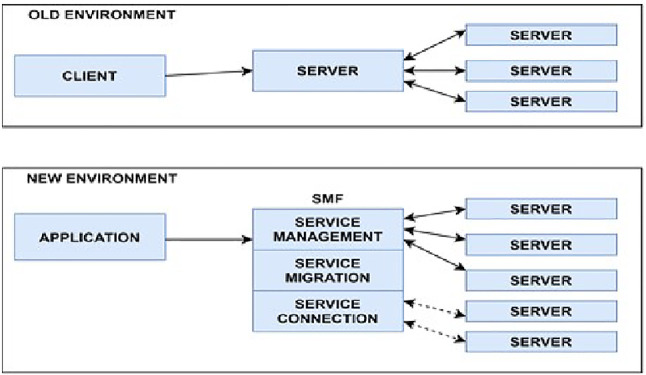
Effect of introducing SMF in the client–server environment

To respond to the security challenges highlighted above, a new approach to delivering services is now required in which it is possible to look at issues of security, deployment, replication, or migration of services on a regional, national, or global scale. To achieve this, a new entity called the Service Management Framework (SMF) has been proposed [30]. The new environment is shown in Fig. 6.

**Fig. 7:**
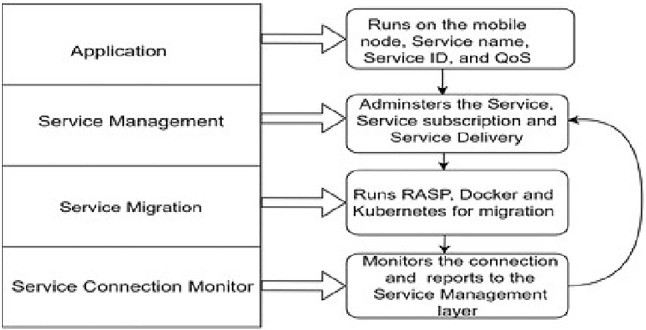
Details of the service management framework

Figure 7 shows the details and interactions of the Service Management Framework.

The SManL ensures an application is assigned to a relevant secure server and is given the correct parameters to securely use that server. SManL, therefore, supports two general interfaces. The first allows a Service Provider to register a service. This is shown in Table 7. The second interface allows an application to request a service. SManL then provides the application with the necessary parameters to contact a server that runs the service. This is shown in Table 8.

**Benefits of service management framework**

The Service Management Framework is a new way of deploying and managing services in distributed environments. It allows clients to find services, and provides communication endpoints and capabilities which allow a reliable session to be developed. It, therefore, increases the security, efficiency, and management of services, and will be a key part of Future Internet architectures.

**Table 7:** Registration service protocol for service providers

Source ->Destination	Type of Message	Actions at Destination
Service Provider ->SManL	Register Service Request [Service name, Service version, Resource requirements (CPU, Memory, Network, Storage), Restriction list, Security level, QoS, Location Restrictions, Maximum Replicas, Actual binary of the service]	SManL first checks to see if the service and version are already registered. If not, it creates a new service structure and populates this structure with data passed by the service provider. It then creates a unique Server ID and a Service Capability.
SManL ->Service Provider	Reply to Register Service Request [Success = 1; Failure = 0; Server ID, Server Capability]	The Service Provider stores the returned parameters

**Table 8:** Request service protocol for applications

Source ->Destination	Type of Message	Actions at Destination
Application ->SManL	Request Service Request [App Node ID, Service name, Service version, QoS Requirements, Node Location, and Network Interfaces]	SManL first looks to see if there is a registered service that meets the request. If a service structure is found, SManL then sees if there already is a server which runs the service close to the application. If there is no server available, then a Cloud System is selected, and a new server is started.
SManL ->Application	Reply to Request Service Request App Node ID, Service name, Service ID, Server Location, QoS Requirements and Server IP address, Service Capability]	The Application uses the Server IP address and Service Capability to contact the server and use the service.

## Towards an implementation framework

**Fig. 8:**
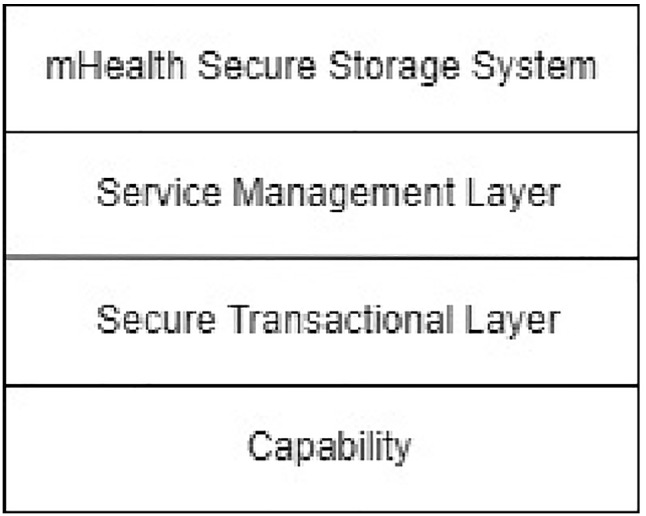
A new practical implementation framework

By combining the three new mechanisms (Service Management Layer, Secure Transactional Layer, and Capability System), it is possible to create a practical implementation framework for secure healthcare environments, as shown in Fig. 8.

### mHealth application storage

A key part of the new framework is the provision of mHealth applications and services for healthcare systems. In terms of required applications and services, there is the need to develop a secure mHealth storage system (as part of the mHealth application) that provides several functions including: create, store, modify, and delete healthcare records.

### The prototype scenario

Generally, healthcare data, which are classed as sensitive data, are collected from patients through wearable devices. These wearable devices communicate with a mobile device through an authorised mHealth application that has been installed. A mobile device receives collected healthcare data from a wearable device. The healthcare data (received) may be collected on a mobile device database and/or transmitted to be stored on a hospital database and Cloud storage. The FUSE/NMS is implemented for a storage, to control and manage healthcare data residing in the system.

All mHealth devices, clients, and servers must be connected to the Service Management Layer (SManL). To be able to use a service, the mHealth application has to send a service request by giving a service name to the SManL to find a suitable server. The SManL then takes the service name (from the request), and scans through a list of services. If there is a valid service for the request, it will return the Service Structure for that service which contains a list of servers that is currently running that particular service. After that, the SManL chooses a server which contains the requested service and contacts the server. The chosen server accepts a request from the SManL, and the SManL then passes an instruction to the mHealth application. The mHealth application can now send a session start request and a service request to the chosen server. The server accepts the requests, so that the mHealth application can use the service. Finally, the mHealth application sends an end request to the chosen server to terminate the session.

**Fig. 9:**
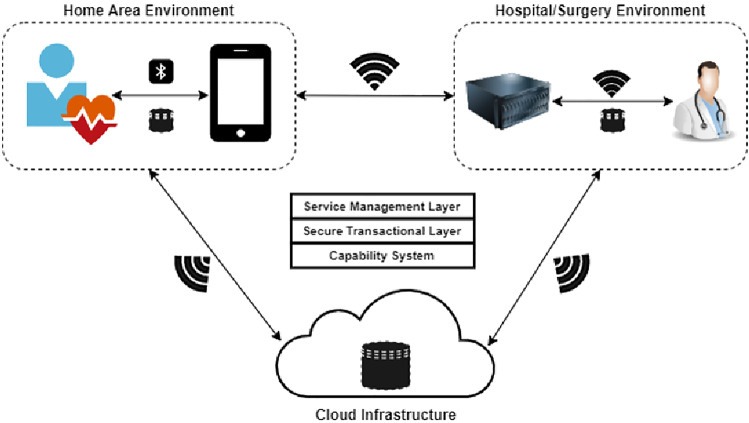
A proposed prototype for a secure healthcare service

The client now initiates the request for connecting to the server through SRPC (see Sect. 4.2) and waits for a response to be returned from the server. Once the connection to the server is successfully established, the client sends encoded instructions to the server where it decodes and processes the data and then returns a response to the client.

Capabilities (see Sect. 4.1) will be used as a main authentication mechanism in the system. Every entity in the system must be represented by a capability. Capabilities manage which entity will be able to gain access to each object in the system as well as identify that the request is, on behalf of which entity. Each entity will be represented using a fixed string which statically assigns to different capabilities. In addition, capabilities are passed in the SRPC call.

The system being developed is shown in Fig. 9.

## Implementation

This section describes in detail the implementation and evaluation of the proposed framework using the relevant technologies.

### The prototype system for mHealth secure storage

**Fig. 10:**
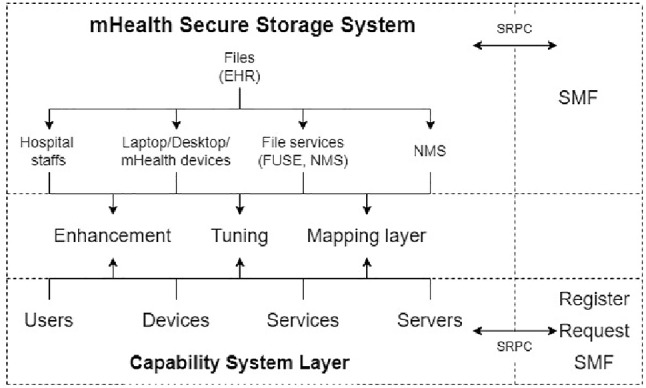
Enhancing an mHealth Secure Storage System

The Capability System Layer is only a basic system which allows the creation of users, devices, and services, and enables servers to be added to implement services. When users, devices, services, and servers are created, only very generic details are assumed. It depends on the system developer to enhance these entities for the particular system. In this case of the mHealth Secure Storage Application, a user could be a doctor, nurse, administrator, and patient. We therefore need to enhance the basic structure provided by the capability library to implement our system. Moreover, once new users, devices, services, and servers are created, they will be registered at Service Management Layer (SMF).

For this healthcare system, users are specified in terms of role, rank, and speciality. For example, a user can be a doctor(role), a consultant (rank), and a dermatologist (speciality).

An example below shows how to enhance the basic capability (see Fig. 3) to be a specific doctor capability.

The structure of doctor capability (CAP_DOCTOR) has presented as below.

- **1. Type field** (8 bits)- Doctor
- **2. Sys field** (4 bits)

- P bit: If set, this user is private. [Set]
- S bit: If set, this user is created by the system. [Set]
- M bit: If set, this user holds a master capability. [Set]
- C bit: If set, this user capability is changeable. [Not set]

- **3. Property field** (12 bits)

- BIT(0)- If set, person has access to EHR. [Set]
- BIT(1)-If set, person can operate/supervise medical equipment. [Set]
- BIT(2) - If set, person can access hospital IT services. [Set]
- BIT(3)-If set, person has access to specialised areas in hospital. [Set]
- BIT(4)-If set, person can order medical tests and drugs/treatment for patients. [Set]
- BIT(5)-If set, person can order that a patient is sent to or discharged from hospital/ward. [Set]
- BIT (6)(7) - Rank (2 bits) - 4 levels (Medical student, Junior doctor, Senior doctor, and Consultant)
- BIT (8)(9)(10)(11) - Specialist (4 bits) - 8 areas (Anesthetist, Emergency medicine, Radiologist, Psychiatry, Surgery, Oncologist, Dermatologist, Haematologist)

- **4. Object ID** (72 bits)
- National Insurance (NI) number
- **5. Random bit field** (16 bits)
- **6. Hash field** (16 bits)

Since the doctor must be able to access to EHR, Table 9 shows an example of doctor access rights based on their ranks and specialists.

**Table 9:** Access to file/EHR based on role, rank, and specialist

Role	Rank	Specialist	File/EHR
Doctor	Medical student	N/A	r–
	Junior doctor	N/A	rw-
	Senior doctor	Anaesthetist	rw-
		Emergency med	rw-
		Radiologist	rw-
		Psychiatry	rw-
		Surgery	rw-
		Oncologist	rw-
		Dermatologist	rw-
		Haematologist	rw-
	Consultant	Anaesthetist	rw-
		Emergency med	rw-
		Radiologist	rw-
		Psychiatry	rw-
		Surgery	rw-
		Oncologist	rw-
		Dermatologist	rw-
		Haematologist	rw-

#### Basic file structure (inode)

**Fig. 11:**
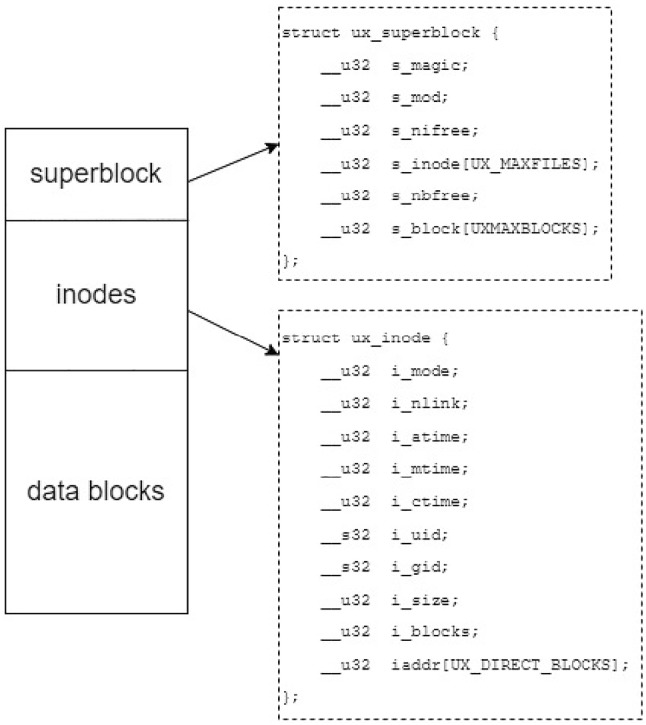
Filesystem structure

In all filesystem, a file is managed using an inode structure. The developed FUSE is implemented as a filesystem (Fig. 11). It contains 512-byte size blocks of data. The filesystem consists of three main elements including superblock, inode, and directory entry which are specified below.

**1. Superblock:** The superblock stores information about the status of inodes and data blocks, which implies an availability of inodes or data blocks.

**2. Inode:** Each file in the system is managed using an inode. In an inode structure, it stores information regarding file objects including:

- Inode number: An inode number of the file. Each file can only have one inode number.
- File type (i_mode): indicates the type of element including Regular file (S_IFREG), or Directory (S_IFDIR)
- Link to location of the file (i_nlink): The number of hard links to the disk block that stores the file’s actual contents
- Timestamp (i_atime, i_mtime, i_ctime): Contains last accessed time, modification time, and (inode) change time
- User ID (i_uid): specifies the owner of the file
- Group ID (i_gid): specifies the group owner of the file
- File blocks (i_addr): an array of a size equal to the number of blocks that the file has been allocated and contains the numbers of the data blocks that are allocated for its use.
- Number of blocks (i_blocks): The number of data block it can be allocated.
- Offset (b_offset): specifies the position within the current data block and it is used by functions to trace the amount of associated data.

**3. Directory entry:** It presents a 32-byte structure in which a 4-byte fields storing the number of inode (d_ino), and a 28-byte field storing the name of file (d_name).

**4. Storage mechanisms:** In general, most data are stored on a hard disk or a solid-state drive (SSD). However, there is now an interest in block network storage called Network Memory Server (NMS). The NMS stores blocks of data in secure random memory (RAM) over the network. Hence, it can be used as persistent storage for filesystems.

#### The implementation system

**Fig. 12:**
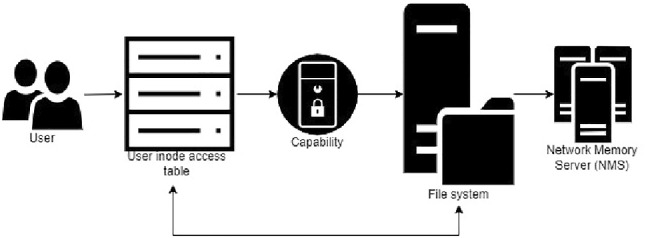
mHealth secure storage system

The implementation of the system is shown in Fig. 12. This combines the user capability system, the filesystem, and the NMS. Each user has three sets of Inode access table which include Public access table, Role/Rank access table, and Private access table. This allows them to access details from the public domain but also the role/rank as well as their private data (e.g., email). To access to the file, the user must access the file based on their access rights provided in their access tables. Each inode access table has a pointer to the capability to verify which access rights the current user has for the file (Read-only, Read/Write, and Execute). The user will not be able to access the file if their access tables do not provide the pointer to capabilities accessing to the file.

When the file is created, the inode and the capabilities of the file are also created. The capabilities of the file include (1) Master capability, (2) Read/Write capability, (3) Read-only capability, and (4) Execute capability. These capabilities are in the structure of the inode.

The structure of EHR capability (CAP_FILE), which considers as a file, has shown as below.

- **1. Type field** (8 bits)-File
- **2. Sys field** (4 bits)

- P bit: If set, this file is private. [Set]
- S bit: If set, this file is created by the system. [Not set]
- M bit: If set, this file holds a master capability. [Not set]
- C bit: If set, this file capability is changeable. [Set]

- **3. Property field** (12 bits)

- BIT(0): If set, Read [Set]
- BIT(1): Read/Write [Set]
- BIT(2): Execute [Set]
- BIT(3): Delete [Set]

- **4. Object ID** (72 bits)

- OBJECT_ID Flags
  $\circ$ Bit 0: If set, the file is a directory.
  $\circ$ Bit 1: If set, the file is an executable file.
- General access type
  $$\circ $$ 00: Public file
  $$\circ $$ 01: Private file
  $$\circ $$ 10: Group file
  $$\circ $$ 11: EHR
- 32-bit IP address of the device that manages the file
- 28-bit inode number of the file on that device

- **5. Random bit field** (16 bits)
- **6. Hash field** (16 bits)

The user who creates the file is considered the owner of the file; therefore, this user is holding the master capability which allows the full access (Read-only, Read/write, and Execute) to the file. Moreover, this user can also create proxy capabilities to allow other users to access to the file.

**Fig. 13:**
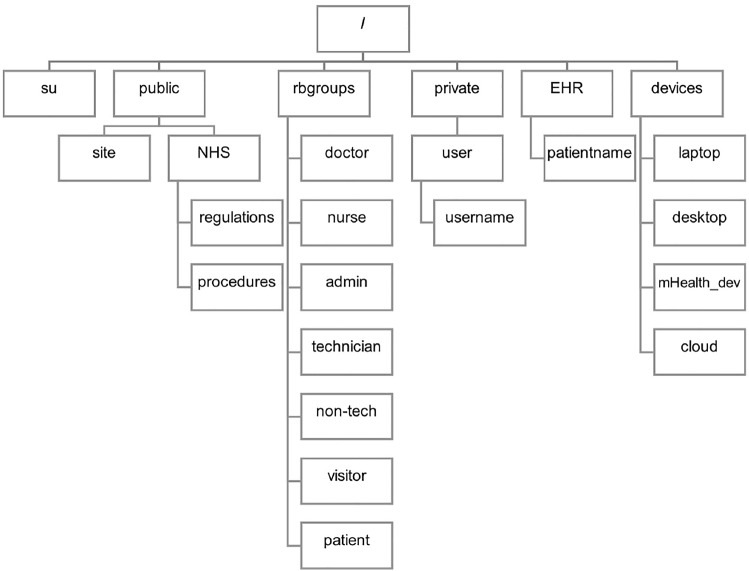
The mHealth secure storage filesystem directory

Figure 13 presents the file directory of the mHealth Secure Storage System. Each directory can be defined as below.

- su: Files for a superuser
- public: Public files which everyone can access
- rbgroups: Files for role-based groups
- private: For users’ private data
- EHR: Electronic Health Record
- devices: Any equipments in the system.

### Evaluation

Initially, the research began with conducting a literature review by identifying, analysing, and comparing different existing studies about security frameworks. Several security frameworks were developed (See sect. 3.1); however, none of existing frameworks provides a full set of security requirements (Confidentiality, Integrity, Availability, Non-repudiation, Authentication, Authorisation, Accountability, Auditability, and Reliability), and all healthcare environment components (User, Devices, Data, IT infrastructure, and Physical space access) at the same time.

To be able to test the functionalities of the proposed framework properly, a prototype combining various developed security mechanisms: (1) Capabilities; (2) SRPC; (3) SMF/SManL; and (4) mHealth secure storage, is developed and stands for the purpose of testing.

To test access rights of users with different capabilities, the mHealth secure storage was developed. The capability system was first developed as a basic system and only provided generic capabilities. Therefore, these basic capability structures were enhanced to be specific to the smart healthcare system. Capabilities do not only provide the user authentication and authorisation, but it also protects unauthorised accesses to devices, digital data, IT infrastructure, and physical space. The test was conducted by verifying the access rights of each user to the filesystem directory (Fig. 13) based on their roles. To access a file or directory in the filesystem, the system first checks if you are from the group that control the directory, then you will be allowed to access to the directory. For example, only doctors, nurses, and admin staff are allowed to directly access EHR records in this system. If someone wants to access an EHR file. The system will first check whether they are doctors (CAP_DOCTOR), nurses (CAP_NURSE), or admin staff (CAP_ADMIN) to allow directly access EHR records. The results confirmed that capabilities provided security requirements of confidentiality, integrity, authentication, and authorisation by only allowing authorised users to access the file directory and preventing unauthorised users from accessing it. Moreover, capabilities also provided security requirements of non-repudiation and accountability, since activities of the capability holder can be tracked in the system.

SRPC was used to encode data transmitted in the system. It also supported the type array. This is an improvement of a traditional RPC as the type array is not passed in the normal RPC call. Additionally, this approach can detect whether the type and value passed are correct. This improves the reliability of the smart healthcare system that must provide a consistent intended of service at all time.

The SMF/SManL provided some basic functions to register users, devices, and services, and add servers to services. Hence, it ensured the availability and reliability of services residing in the system, recorded activities, improved efficiency, security, as well as management of services.

Despite the fact that the prototype may contain less numbers of layer; nevertheless, the evaluation presented that the implemented prototype that consists of 4 layers: Capability system, SManL/SMF, SRPC, and mHealth secure storage, developed in this research provided essential security requirements required by smart healthcare systems as well as a practical protection for users, devices, digital data, IT infrastructure, and physical areas that are main assets as discussed in Sects. 2 and 3.2.

Table 9 shows how the prototype provides the security requirements for healthcare environments.

**Table 10:** How the proposed framework achieves security requirements

Security requirements	(1)	(2)	(3)	(4)
Confidentiality	*			*
Integrity	*			*
Availability			*	*
Non-repudiation	*			
Authentication	*			*
Authorisation	*			*
Accountability	*		*	
Auditability			*	
Reliability	*	*	*	*

Table 11 shows how the prototype provides practical security for users, devices, digital data, IT infrastructure, and access to physical space.

**Table 11:** How the proposed framework achieves security for healthcare environment components

Protection	(1)	(2)	(3)	(4)
Users of the system	*			
Device and home access	*		*	
Digital data	*	*	*	*
IT infrastructure	*	*	*	
Access to physical space	*			

## Conclusions and future work

This research addresses several issues regarding securing electronic health data, which integrates modern electronic hospital systems which are being distributed both on site and remote, along with mHealth endpoints those involve various related stakeholders including patients, healthcare professionals, and other hospital staff.

In this paper, healthcare environments components were identified, and can be divided into five categories: users of the system, devices and home access, digital data, Hospital infrastructure, and access control of physical sites. Therefore, it is necessary that these components must be protected.

This paper focused on building an architecture for securing future healthcare environments. Using an implementation framework combining various new security mechanisms to secure healthcare environments as well as other domains, a prototype was developed. The prototype provides a complete set of requirements for end-to-end security and makes a novel contribution to information security, especially in the context of healthcare environments.

The system obeys the properties of secure healthcare systems as defined by our comprehensive analysis. However, this paper also shows how this can be practically implemented to build secure intelligence environments.

While it opens new insights for research in the security of healthcare environment areas, therefore, it can be extended to many more directions for future research.

Although, the result showed that the implemented prototype improved the security of smart healthcare systems. However, the test was conducted on the mHealth secure storage system that was developed in this research to provide some basic functionalities of the hospital filesystem. Therefore, this implemented prototype needs to be tested and integrated into small healthcare institutions (e.g., GP surgery, clinic, and small hospital), and then large healthcare institutions (e.g., a large NHS hospital).

Moreover, new technologies such as Artificial Intelligence (AI) and Machine Learning (ML) can be introduced as new security mechanisms for the proposed information security framework. Not only the AI/ML can enhance diagnostics, patient care, and clinical decision support across the medical service. It can also be used to analyse the data flow within smart healthcare systems to detect what are “normal” or “abnormal” behaviours of each user, device, and service in the system, so that it can protect smart healthcare systems from cyberattacks such as ransomware.
